# Supplementary material for: Identification of Nordic Berries with Beneficial Effects on Cognitive Outcomes and Gut Microbiota in High-Fat-Fed Middle-Aged C57BL/6J Mice
Source: Nutrients. 2022 Jun 30;14(13):2734. doi: 10.3390/nu14132734 (PMC9269296; doi:10.3390/nu14132734)
Supplement: Supplementary file 1 [file nutrients-14-02734-s001.zip › nutrients-1735617-supplementary.pdf]

nutrients

MDPI

Supplementary Materials

Identification of Nordic Berries with Beneficial Effects on Cognitive Outcomes and Gut Microbiota in High-Fat-Fed Middle-Aged C57BL/6J Mice

Fang Huang [1,2,*,†], Nittaya Marungruang [3,†], Olha Kostiuchenko [4,5], Nadiia Kravchenko [4,5], Stephen Burleigh [4], Olena Prykhodko [4], Frida Fåk Hållenius [4] and Lovisa Heyman-Lindén [3,6]

Table S1. Composition of diets. All diets were designed to have an equal caloric content of fat, protein, and carbohydrates (including glucose, fructose, and sucrose).

	LF	HF	Lingonberries	Bilberries	Blackcurrants	Cloudberries	Seabuckthorn	Blueberries
Calculated energy(kcal)								
Protein	708.0	708.0	708.0	708.0	708.0	708.0	708.0	708.0
Carbohydrate	2840.0	815.2	815.2	815.2	815.2	815.2	815.2	815.2
Sucrose	195.2	195.2	195.2	195.2	195.2	195.2	195.2	195.2
Fructose	64.0	64.0	64.0	64.0	64.0	64.0	64.0	64.0
Glucose	64.0	64.0	64.0	64.0	64.0	64.0	64.0	64.0
Fat	405.0	2430.0	2430.0	2430.0	2430.0	2430.0	2430.0	2430.0
Fiber	0.0	0.0	0.0	0.0	0.0	0.0	0.0	0.0
Other	0.0	0.0	0.0	0.0	0.0	0.0	0.0	0.0
Total kcals	3953	3953	3953	3953	3953	3953	3953	3953
Calculated energy per gram diet (kcal/g)								
kcal/g	3.7	5.1	5.1	5.1	5.1	5.1	5.1	5.1
Calculated energy (kcal%)								
Protein	18	18	18	18	18	18	18	18
Carbohydrate	72	21	21	21	21	21	21	21
Fat	10	61	61	61	61	61	61	61
Fiber	0	0	0	0	0	0	0	0
Fiber content (g/100 g diet)								
Total fiber[1]	4.7	6.5	6.1	6.4	6.6	7.0	6.1	6.5

[1] Fiber coming from added cellulose (LF and HF diets) or added cellulose plus fibers present in berries (berry diets) added to HF diet at 6% (w/w) dry weight basis.

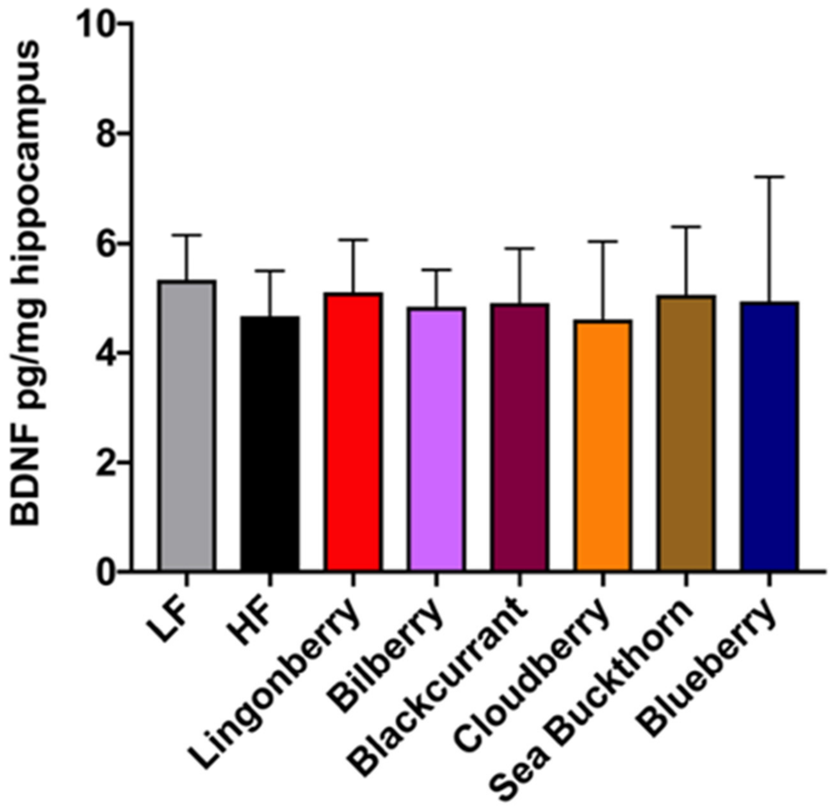

Figure S1. BDNF (pg/mg) level in hippocampus. The brain homogenates were made on different weights of hippocampus.

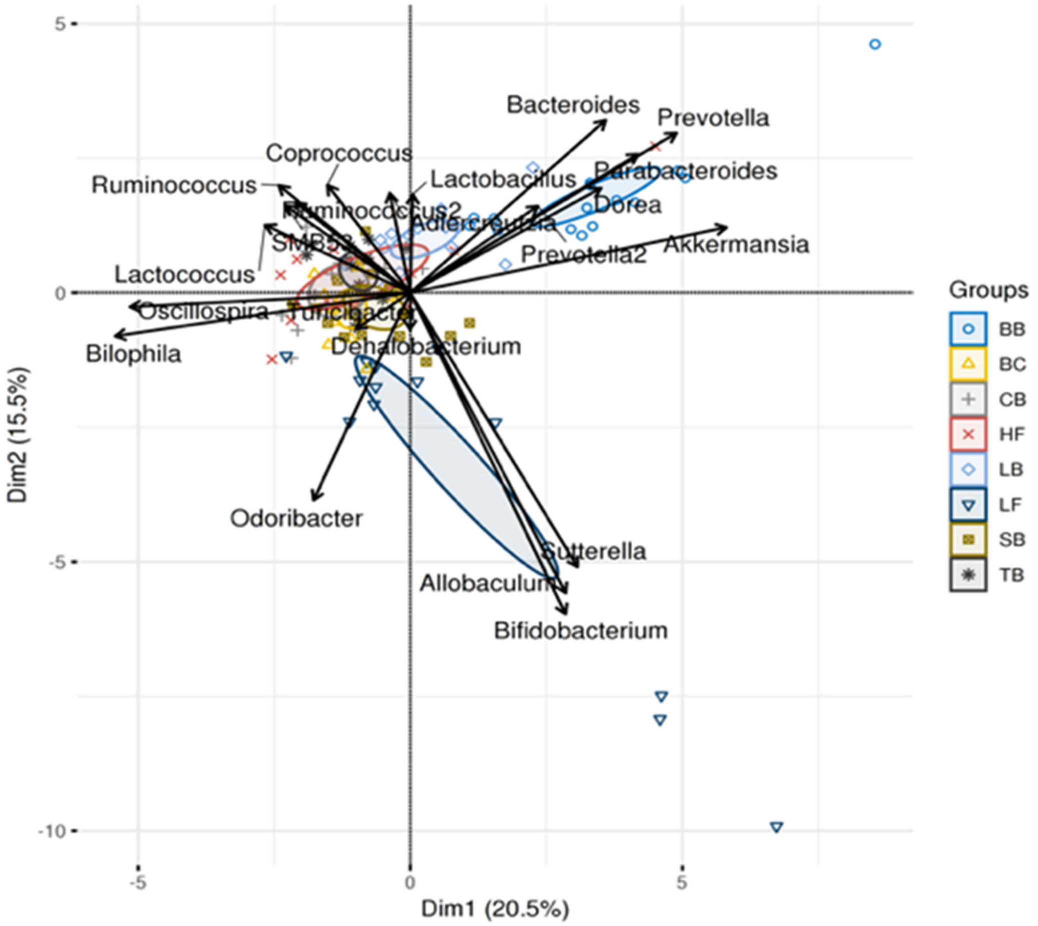

Figure S2. Principal component analysis (PCA) plot of the gut microbiota at genus level in mice fed LF, HF, lingonberry, bil-berry, blackcurrant, cloudberry, sea buckthorn and blueberry.
